# Study on Hydrolytic Degradation of Polyester and Polyamide in Basic Solutions at High Temperatures

**DOI:** 10.3390/polym17233090

**Published:** 2025-11-21

**Authors:** Haotian Fan, Haibo Wang, Zhiyuan Tian, Jiaxin Shi, Wei He, Duo Qi, Baohua Guo, Jun Xu

**Affiliations:** 1 College of Materials Science and Engineering, Shenyang University of Chemical Technology, Shenyang 110142, China; fanhaotian24@163.com (H.F.); tianzhiyuan@ekaiming.com (Z.T.); hw@syuct.edu.cn (W.H.); qiduo@syuct.edu.cn (D.Q.); 2 Advanced Materials Laboratory of Ministry of Education (MOE), Department of Chemical Engineering, Tsinghua University, Beijing 100084, China; shijx13@tsinghua.org.cn (J.S.); bhguo@tsinghua.edu.cn (B.G.); 3 SINOPEC Research Institute of Petroleum Engineering Co., Ltd., Beijing 102206, China; wanghb.sripe@sinopec.com

**Keywords:** polyester, polyamide, hydrolysis mechanism, accelerated degradation

## Abstract

Plugging materials play a crucial role in oil production. Current development of temporary plugging agents still faces challenges such as insufficient sealing strength, poor temperature resistance, and complex manufacturing processes. To enable barrier materials for higher-temperature applications, we extensively studied the hydrolysis processes of aliphatic polyesters, polyamides, and thermosetting resins at different temperatures, analyzing the hydrolysis mechanisms of representative poly (butylene terephthalate) (PBT) and polyamide 6 (PA6). Inspired by the etching effect inspired by wood decay under humid environments, PBT was selected as the continuous phase to design and prepare a PBT/PA6 blend exhibiting physicochemical synergistic effects. The results indicate that PBT/PA6 blends exhibit significantly faster hydrolysis characteristics at 120 °C compared to pure thermoplastic polyesters. We found this to be the result of a synergistic effect where the terminal amine groups released during PA6 hydrolysis catalyze PBT hydrolysis, while the etching effect of the PBT continuous phase during hydrolysis accelerates PA6 hydrolysis. This study innovatively integrates physical–chemical synergistic effects, proposing a material design strategy that significantly enhances degradation rates.

## 1. Introduction

Oil well plugging materials are used to plug highly permeable layers to reduce water seepage in oil wells, which are crucial for increasing crude oil production. Temporary plugging agent is a substance that temporarily reduces the reservoir permeability or temporarily seals off highly permeable layers. It can be mixed with water-soluble substances and injected into the reservoir to rapidly form a thin and dense temporary plugging zone under the action of pressure differential, which can be manually or self-unplugged after a period of time [1]. As petroleum exploration environments become increasingly complex and technical challenges increase, higher requirements are placed on the comprehensive performance of temporary plugging agent materials.

To meet the needs of oil field development, the research and development of degradable temporary plugging agents have become increasingly important. The core component of traditional biodegradable plugging agents is aliphatic polyester, which undergoes hydrolysis under high-temperature conditions. Although PGA-PLA modified materials were tested in the Barnett Shale oilfield in the United States as early as 2011 [2], these materials exhibit rapid hydrolysis rates and mechanical property degradation significantly outpacing material degradation. Consequently, researchers have consistently focused on developing plugging materials with more controllable environmental tolerance. For instance, Liu et al. [3] encapsulated a PLA core within a P(AM/MM/AMPS) copolymer shell to delay PLA degradation and maintain mechanical stability. Alternatively, PLA itself has been modified to address challenges in deepwater drilling. Other researchers have engineered chemical structures to regulate high-temperature hydrolysis rates, such as coupling epoxy resins [4] or hydrophilic segments [5] to accelerate or delay sealant degradation. However, these approaches often suffer from high costs or excessively rapid hydrolysis. Research on fiber [6,7,8], gel [9,10], and starch-based [11,12,13] plugging agents also exists. However, practical application is hindered by issues such as fiber dispersion difficulties and agglomeration blockage, gel water absorption accumulation blockage, and excessively long degradation cycles for starch. Therefore, achieving controlled degradation of plugging materials through low-cost raw materials combined with physical and chemical structural design has become one of the critical challenges requiring urgent solutions in this field [14].

This established a preliminary material platform library capable of regulating degradation rates across a broad range. While slow-degrading materials are readily available, certain applications require rapid degradation. To further broaden the range of degradation rates, we drew inspiration from the natural decay process of wood. By leveraging the synergistic “etching acceleration-chemical catalysis” interaction between poly (butylene terephthalate) (PBT) and polyamide 6 (PA6), we achieved a degradation rate where 1 + 1 > 2. Specifically, although PA6 hydrolyzes slowly, the resulting terminal amine groups catalyze the hydrolysis of PBT. The rapid hydrolysis of PBT induces etching within the polymer matrix, creating numerous pores that increase the specific surface area. This, in turn, significantly accelerates the hydrolysis rate of PA6. Through this synergistic effect, the degradation rate of the blend material is markedly enhanced. This study proposes a material design strategy based on physicochemical synergistic effects that can significantly enhance degradation rates. It provides crucial reference for the design and application of high-temperature temporary plugging agents.

## 2. Sample Preparation and Methods

### 2.1. Materials, Chemicals and Reagents

Polybutylene terephthalate (PBT, type TH6100, with intrinsic viscosity 0.85 dL/g) was purchased from Xinjiang Blue Ridge Tunhe Science and Technology Co., Ltd., (Tunhe, China). Polyethylene terephthalate (PET, type CZ-318, with intrinsic viscosity 0.84 dL/g) was purchased from Sanfame Group Co., Ltd., (Wuxi, China) Polyamide 6 (PA6, type YH400, MFR = 15 g/10 min) was purchased from Sinopec Baling Petrochemical Co. (Yueyang, China). Polyamide 66 (PA66, type 101 L, MFR = 50 g/10 min) and Polyamide 1010 (PA1010, type LC1200, MFR = 240 g/10 min) was purchased from Dupont China Holding Co., Ltd. (Shenzhen, China). Epoxy resin (E51, E44) was purchased from Sinopharm Chemical Reagent Co., Ltd., (Shanghai, China). NaOH, AR, Shanghai Macklin Biochemical Technology Co., Ltd., (Shanghai, China). Ethanol, AR, Shanghai Titan Scientific Co., Ltd., (Shanghai, China). All materials were used as received without further purification.

### 2.2. Apparatus

400 ML Hydrothermal aging tanks was purchased from Fann Instrument Company (Houston, TX, USA); parallel twin-screw extruders (screw diameter of 16 mm), the HAAKE Rheomex PTW 16 and Fourier-Transform Infrared Spectroscopy (FT-IR) Nicolet-6700 were purchased from Thermo Fisher Scientific (Waltham, MA, USA); Gas Chromatography–Mass Spectrometer (GC-MS) and Differential Scanning Calorimetry (DSC) were purchased from Shimadzu Company (Kyoto, Japan); Scanning Electron Microscopy (SEM), VEGA 3 SBH was purchased from Tescan Group (Brno, Czechia); the constant temperature drying oven ED720 was purchased from BINDER GmbH (Baden-Wurttemberg, Germany); Liquid Nuclear Magnetic Resonance Spectrometer (^1^H-NMR) was purchased from JEOL Ltd., (Akishima, Japan).

### 2.3. Preparation of Pure Polymer Pellets

PBT, PET, PA6, PA66, PA1010 pellets with the diameter of about 3 mm were dried in 80 °C vacuum oven for 24 h. According to the proportion of weighing, the epoxy resin E44 (or E51) and adipic diamine (weight ratio of 100/14) were mixed and cured at 120 °C for 1 h. The curing thermosets was crushed to obtain epoxy pellets.

### 2.4. Preparation of Blend Pellets

The dried PBT and PA6 were melt blended in a twin-screw extruder with a certain mass ratio (70/30, 80/20). The temperatures in heating zones 1–6 were 200 °C, 220 °C, 240 °C, 240 °C, 240 °C, 240 °C, and the temperature of the head was 230 °C, and the speed of the screw was 60 r/min, and the feeding frequency was 1 Hz. The extruded blend filaments were pelletized to produce uniformly sized PBT/PA6 cylindrical pellets with a length of approximately 3.5 mm and a diameter of approximately 2 mm.

### 2.5. Weight Loss Analyses

Different samples with an initial mass (*m*_0_) of 5 g were added to an aging vessel containing 350 mL of a NaOH aqueous solution with pH of 10. The aging jars with different samples were put into a constant temperature drying oven at 120–180 °C, and the samples were taken out every 2 days and washed with anhydrous ethanol and then dried to a constant weight to record their weights (*m*). The percentage of weight loss (*R_d_*) was calculated:

$$R_{d}=\frac{m_{0}-m}{m_{0}}\times 100\%$$

where *m*_0_ is the mass of the sample before degradation and *m* is the mass of the sample after a certain time of hydrolytic degradation.

### 2.6. Differential Scanning Calorimetry (DSC) Measurements

The DSC thermograms of the samples were tested by heating 3–5 mg of the samples sealed in a crucible under nitrogen atmosphere from room temperature to 350 °C at a rate of 10 °C/min.

### 2.7. Fourier-Transform Infrared Spectroscopy (FT-IR)

The FT-IR spectra of the hydrolyzed samples were determined using the KBr compression method and transmission mode with a wave number range of 400–4000 cm^−1^, a resolution of 4 cm^−1^ and a scan number of 32.

### 2.8. Liquid Nuclear Magnetic Resonance Spectrometer (^1^H-NMR)

A certain mass of the sample before and after hydrolysis was dissolved and subjected to nuclear magnetic resonance spectroscopy (^1^H-NMR) measurement using trifluoroacetic acid (TFA) as solvent.

### 2.9. Gas Chromatography–Mass Spectrometer (GC-MS) Measurements

The filtered liquid phase product was subjected to gas chromatography–mass spectrometry (GC-MS) testing, 30 mm × 0.25 mm × 0.25 μm (TR-5) flexible quartz capillary columns were used and the carrier gas was high-purity helium at a flow rate of 1 mL/min. Inlet temperature was 280 °C and detector temperature was 280 °C. The column temperature was 60 (3 min) -250 °C with the heating rate of 15 °C/min. The ion source is electron ionization source.

### 2.10. Scanning Electron Microscopy (SEM) Measurements

Dumbbell-shaped strips were injection molded from PBT/PA6 (7/3) blend samples using an injection molding machine. Portions of these strips underwent weight loss tests at 120 °C and 150 °C for 48 h. Specimen sections before and after hydrolysis were subjected to brittle fracture testing in liquid nitrogen. Following 60 s of gold sputtering at 8–10 mA, fracture surfaces were examined using scanning electron microscopy (SEM) at magnifications of 2000× and 5000×.

## 3. Results and Discussion

Temperature affects the rate of molecular thermal motion, e.g., elevated temperatures accelerate polymer chain movement, promoting hydrolysis and catalytic processes, while lower temperatures inhibit chain motion, significantly slowing degradation rates. Different types of polymers exhibit varying degrees of temperature sensitivity, making it essential to study hydrolysis behavior at different temperatures.

**Figure 1:**
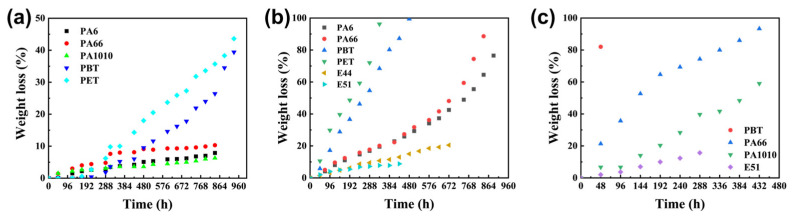
Hydrolysis of several polymers at different temperatures. (**a**–**c**) show the hydrolysis curves of polymers at 120, 150, and 180 °C, respectively.

The hydrolysis rates of aromatic polyesters, polyamides, and epoxy resins were evaluated at different temperatures, with results shown in Figure 1a–c. Epoxy resin forms a three-dimensional crosslinked network with ether bonds (-O-) in the main chain after curing. It lacks readily hydrolysable groups and possesses a dense crosslinked structure, resulting in negligible hydrolysis. Even at elevated temperatures, the crosslinked network continues to block water molecule penetration. The high stability of ether bonds also maintains extremely slow degradation [15]. The amide bonds (-CONH-) in the polyamide backbone exhibit higher stability than ester bonds due to the interaction of the nitrogen atom’s lone pair electrons with those of the carbonyl group. Additionally, strong intermolecular hydrogen bonds in polyamide cause segments to stack tightly. At 120 °C, water molecules can only weakly overcome the amide bond energy barrier and struggle to penetrate internally, resulting in slow surface hydrolysis [16,17]. As temperature increases, some hydrogen bonds break, expanding interchain gaps. This enhances water penetration and bond cleavage efficiency, accelerating hydrolysis. However, the conjugated structure of amide bonds still results in slower rates than polyester. The ester bonds (-COO-) in aromatic polyester main chains exhibit high polarity and low activation energy, allowing the hydrolysis energy barrier to be overcome at 120 °C. As the molecular chain spacing expands with increasing temperature, water molecule penetration efficiency improves, facilitating hydroxyl group attack on the electron-deficient carbonyl carbon [18]. Consequently, significant hydrolysis occurs at 120 °C. At elevated temperatures of 150 °C and 180 °C, enhanced molecular thermal motion further accelerates bond breaking and water diffusion, leading to a dramatic increase in hydrolysis rate. In summary, polyamide and polyester are more susceptible to temperature variations, offering greater potential for designing their degradation curves. Consequently, these two materials were selected as research subjects for further analysis.

**Figure 2:**
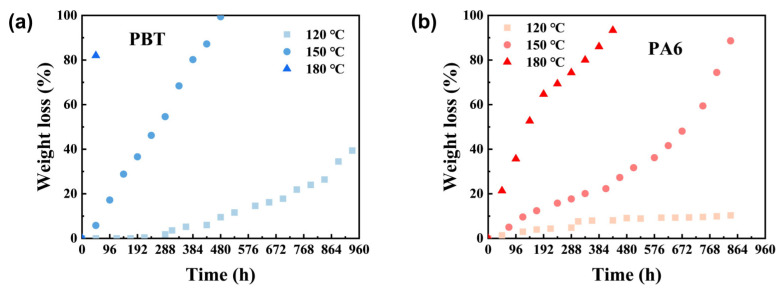
(**a**,**b**) show the weight loss plots for PBT and pa6 at different temperatures.

As shown in Figure 2a,b, the degradation rate of PBT and PA6 materials increases faster with the rise in temperature. The weight loss of PBT is still less than 50% after hydrolysis at 120 °C for 960 h, and it can be completely degraded after hydrolysis at 150 °C for 400 h. The weight loss of PBT reaches more than 80% after hydrolysis at 180 °C for 48 h. In contrast, the hydrolysis of PA6 at 120 °C is very slow and the weight loss after 300 h remains about 10% after 800 h, while the weight loss rate reaches more than 90% after 432 h at 150 °C or 816 h at 180 °C. It is clear that the degradation rate of all the materials accelerates with increasing temperature. However, temporary blocking materials are typically used at fixed temperatures during actual application. Therefore, accelerating material degradation rates through temperature elevation is impractical for real-world application. Consequently, modifying the material itself is necessary to achieve faster degradation speeds.

Traditional views hold that degradation occurs in amorphous regions, making it possible to alter degradation rates by increasing crystallinity through annealing without chemical modification [19]. However, in practical applications, the degradation temperature is not significantly different from the melting point or crystallization temperature of materials like poly (butylene terephthalate) (PET), PBT, and PA6. Consequently, spontaneous annealing may occur during degradation, rendering pre-annealing treatment ineffective. To demonstrate this process, the following research was conducted.

The initial crystallinity of the samples was improved by annealing of PET and verified by DSC test. Figure S1 shows the DSC curves of the PET samples before and after annealing at 180 °C. The melting enthalpy increases, which indicates that the crystallinity of the annealed PET has been improved. Then the degradation rate of the samples before and after heat treatment was tested, as given in Figure S2. However, it was found that the degradation rate of annealed PET was almost the same as that of untreated PET.

The melt enthalpy and crystallinity before and after hydrolysis of different PET samples are shown in Table S1. The DSC curves of PET at different heat treatment levels and hydrolysis times are shown in Figure S3. The melting enthalpy of untreated PET after degradation is almost the same as that of annealed PET. We suggest that non-treated PET will further crystallize in the process of high temperature hydrolysis. The crystallinity of the untreated PET has increased substantially after 24 h of hydrolysis, which is close to that of the PET annealed at 180 °C for 24 h. After short-term hydrolysis, the untreated PET as well as the annealed PET show depressed melting point, which may be caused by the hydrolysis of the amorphous chains connecting the lamellar crystals. On the other hand, the crystallinity of the annealed PET after hydrolysis is similar to that of the untreated PET after short-term hydrolysis, but both are slightly higher than that of the annealed sample. This proves that the enhancement of the crystallinity of PET by heat treatment is limited and this effect is quickly leveled off by the hydrothermal conditions; therefore, it is impossible to regulate the hydrolysis rate of polyester by altering its initial crystallinity through annealing, so other methods are required for further control.

**Figure 3:**
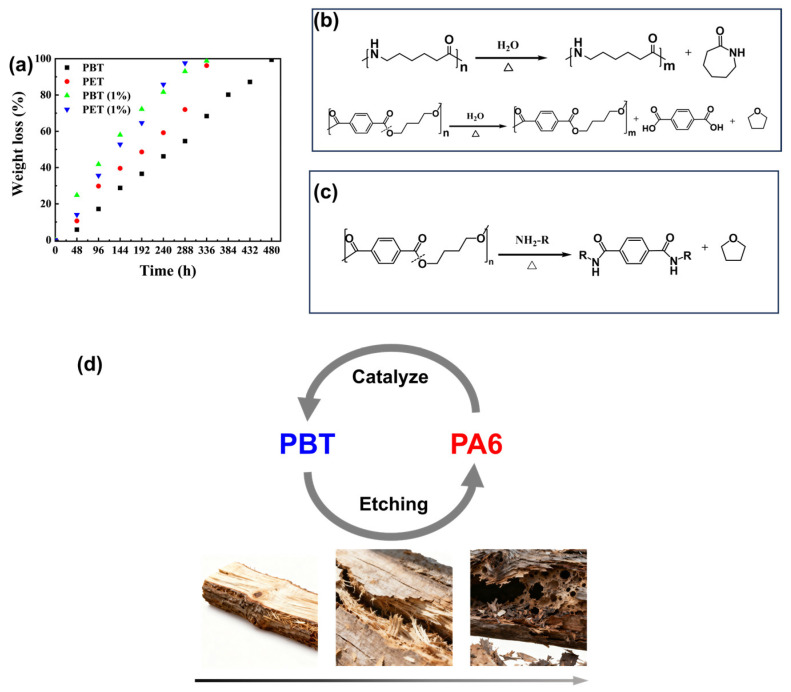
(**a**) shows the weight loss plots of polyesters with organic amines added at 150 °C. (**b**) shows the hydrolysis mechanisms of PBT and PA6. (**c**) shows the reaction mechanism of the ammonolysis properties of polyester. (**d**) shows the design approach for PBT/PA6 blends inspired by the synergistic Etching-Aminic catalysis mechanism inspired by wood decay processes.

Theoretically, amination of polyesters can accelerate degradation [20]. Therefore, we introduced small-molecule organic amines to verify their effect on polyester hydrolysis rates. As shown in Figure 3a, adding 1% (*w*/*w*) hexamethylenediamine to a 150 °C NaOH aqueous solution significantly accelerated the degradation rates of both PBT and PET throughout the entire degradation process. However, in practical applications, small-molecule additives may exhibit migration under high-temperature and high-pressure conditions, potentially compromising performance stability.

**Figure 4:**
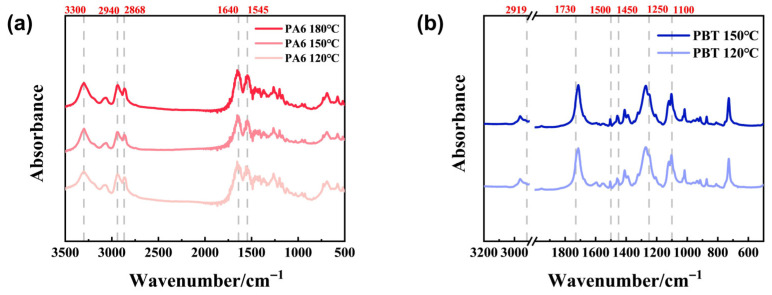
(**a**) shows FT-IR spectra of solid-phase degradation products from PA6 at different hydrolysis temperatures. (**b**) shows FT-IR spectra of PBT solid-phase degradation products at different hydrolysis temperatures.

FT-IR analysis of the solid-phase hydrolysis products of PA6 and PBT at different temperatures is shown in Figure 4a,b. Analysis of the solid-phase degradation products of PA6 reveals the following peaks: 1545 cm^−1^ corresponds to the N-H bending vibration of amide groups and the C-N stretching vibration; 1640 cm^−1^ represents the C=O stretching vibration; 2868 cm^−1^ corresponds to the symmetric C-H stretching vibration peak, 2940 cm^−1^ to the antisymmetric C-H stretching vibration peak, and 3300 cm^−1^ to the amide bond N-H stretching vibration peak. At different temperatures, the positions and intensities of the main peaks showed no significant changes, indicating the uniqueness of the solid-phase degradation products of PA6 at different temperatures. Analysis of PBT degradation solid-phase products revealed absorption bands at 2919 cm^−1^ and 2853 cm^−1^ corresponding to C-H stretching vibrations. The absorption peak around 1730 cm^−1^ originates from C=O vibrations in the ester group. Absorption peaks at 1500–1450 cm^−1^ originate from the vibration of the benzene ring skeleton, while those at 1250 cm^−1^ and 1100 cm^−1^ result from C-O vibrations. Similar to the solid-phase degradation products of PA6, the positions and intensities of the main peaks did not show significant changes at different temperatures, indicating that the solid-phase degradation products of PBT are unique at different temperatures.

**Figure 5:**
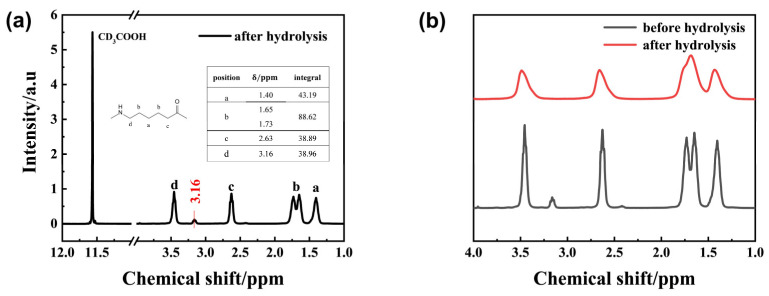
(**a**) shows the ^1^H-NMR spectrum of solid-state PA6 hydrolysis products, with the red number 3.16 in the figure indicating the chemical shift value of the new peak appearing after hydrolysis. (**b**) shows the comparison of ^1^H-NMR spectra of solid-state PA6 hydrolysis products before and after hydrolysis.

Further ^1^H-NMR spectroscopy of the solid-phase degradation products of PA6 is shown in Figure 5a,b. It reveals that the peak positions of the hydrolyzed PA6 sample were similar to those of the unhydrolyzed PA6. The hydrogen proton on the methylene group at the intermediate position within the repeating unit was located near 1.40 ppm, while the hydrogen protons on the two adjacent methylene groups were located near 1.65 ppm and 1.73 ppm, respectively. The hydrogen protons on the methylene groups connected to the C=O group were located near 3.54 ppm. 1.73 ppm, respectively. The hydrogen proton on the methylene group connected to the N-H bond was located near 2.65 ppm, while the hydrogen proton on the methylene group connected to the C=O group was near 3.54 ppm. The peak morphology changed from a relatively low and broad peak to a narrower and more elongated peak, and the peaks of the two methylene groups connected to the central methylene group showed a distinct splitting. This primarily results from PA6 hydrolysis breaking the amide bonds in the molecular chain, generating oligomers that disrupt chain continuity and cause chemical shift deviations. Additionally, a new peak appears at 3.15 ppm in hydrolyzed PA6. Integrating the four main peaks yields an area ratio of approximately 1:2:1:1. However, the content of methylene groups connected to amide groups slightly decreased. This is because the C-N bond breaks during PA6 hydrolysis, forming terminal amine and carboxyl groups. The increased amine content leads to a weaker peak at 3.15 ppm, corresponding to the hydrogen proton in the amine group. This further demonstrates that hydrothermal degradation of PA6 produces a certain number of oligomers bearing terminal amine groups.

Mass spectrometry analysis of the liquid-phase degradation products from PA6 and PBT is shown in Figures S4 and S5. The PBT liquid-phase product is primarily tetrahydrofuran (THF), indicating that 1,4-butanediol readily undergoes cyclization at elevated temperatures to form THF. During PA6 degradation, besides generating oligomers containing terminal amino groups, a large fraction undergoes self-cyclization to form caprolactam [21]. This reduces the number of terminal carboxyl groups, contributing to stability during pH shift toward alkalinity. Analysis of the products reveals that the hydrolysis mechanism of PA6 and PBT is shown in Figure 3b. Furthermore, consistent with the mechanism where small-molecule organic amines accelerate PBT ester bond cleavage, the terminal amino-containing oligomers or monomers generated from PA6 degradation also undergo the reaction depicted in Figure 3c. This promotes PBT ester bond cleavage, thereby accelerating the hydrolysis process of PBT.

A blend-accelerated degradation strategy inspired by wood decay was proposed, as shown in Figure 3d. When wood is exposed to humid environments, microorganisms (such as fungi and bacteria) and insects initially attack readily degradable components like hemicellulose. Once these continuous matrix materials begin to break down, previously protected cellulose fibers become exposed and more susceptible to hydrolysis, oxidation, and microbial attack, thereby accelerating the decay process. Significant degradation of intact cellulose leads to a rapid decline in wood strength, ultimately resulting in a swift deterioration of structural integrity [22].

**Figure 6:**
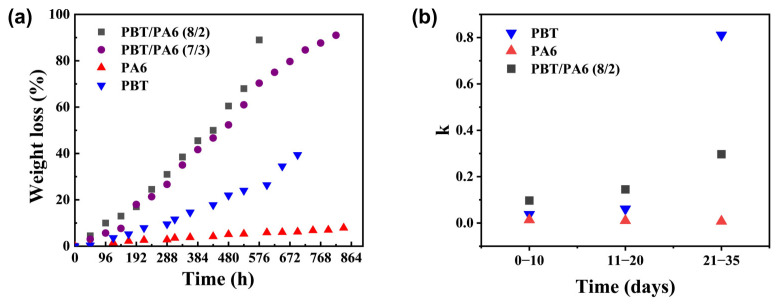
(**a**) shows the weight loss plots for the blends of PBT/PA6 (7/3) and PBT/PA6 (8/2) at 120 °C. (**b**) shows the slope k value of linear fit for polymers across different degradation time periods.

Based on the aforementioned design concept, a PBT/PA6 blend was prepared with hydrolysable PBT as the continuous phase and hydrolytically stable PA6 as the dispersed phase. By varying the PBT content, PBT/PA6 (7/3) and PBT/PA6 (8/2) blends were synthesized to investigate their degradation behavior under hydrothermal conditions at 120 °C. The results are shown in Figure 6a. The degradation rates of both PBT/PA6 blend formulations were significantly faster than those of pure PBT and PA6 materials. The degradation curves for the two pure materials and the PBT/PA6 blends were linearly fitted for the pre-hydrolysis phase (0–10 days), mid-hydrolysis phase (11–20 days), and late-hydrolysis phase (21–35 days). The resulting slope k values are shown in Figure 6b, with the R^2^ values for the fits listed in Table S2. All data exhibited R^2^ values > 85%, confirming the reliability of the data. The accelerated degradation rate of the blend during the early-to-mid hydrolysis phase exceeded that of the two pure polymers, indicating a catalytic effect significantly enhancing degradation. Combining this with the aforementioned mechanism analysis of PA6 terminal amine groups catalyzing PBT hydrolysis, it is reasonable to conclude that this effect arises from PA6 hydrolysis products primarily being terminal amine-containing oligomers. These terminal amine groups catalyze further degradation of PBT, significantly accelerating the overall degradation rate. As hydrolysis progressed into the late stage, the degradation acceleration of the PBT/PA6 blend fell between that of the two pure polymers. Even after the degradation percentage of PBT/PA6 (7/3) and PBT/PA6 (8/2) reached 70% and 80%, respectively, their degradation rates remained higher than that of pure PA6, indicating that catalytic acceleration is not the sole mechanism at work. To investigate this, we studied the macroscopic morphological changes in the PBT/PA6 blends before and after hydrolysis.

**Figure 7:**
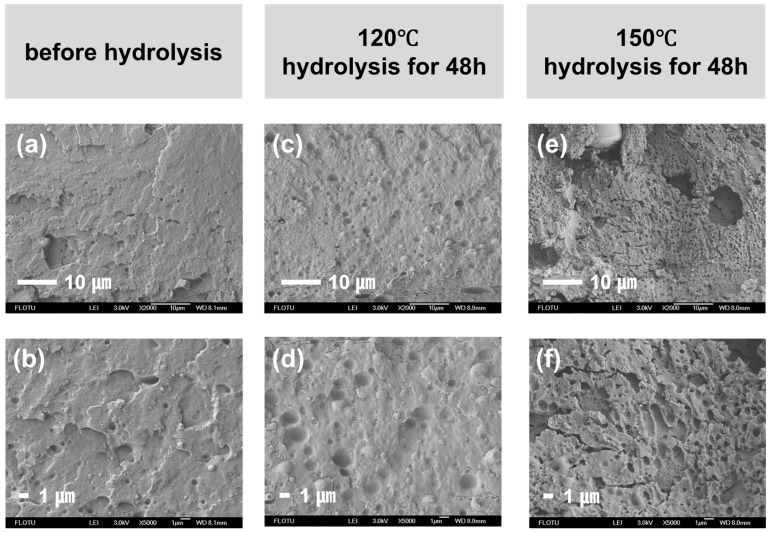
SEM of PBT/PA6 (7/3) blends before and after hydrolysis. (**a**,**c**,**e**) are the SEM image at 2000× magnification. (**b**,**d**,**f**) are the SEM image at 5000× magnification.

Taking the PBT/PA6 (7/3) blend as an example, its scanning electron microscopy (SEM) characterization results are shown in Figure 7. When PBT serves as the continuous phase, Figure 7a,b clearly reveals a distinct “sea-islands structure” in the pre-hydrolyzed blend [23,24]. The chemical differences between the two materials result in weak interaction capabilities, making complete mixing at the molecular level difficult [25]. As hydrolysis progresses, the etching effect becomes pronounced in Figure 7c,d, with the PBT continuous phase forming extensive honeycomb-like voids. Deep hydrolysis of PBT causes severe defects in the blend. Moreover, as shown in Figure 7e,f, these defects become increasingly pronounced with rising hydrolysis temperatures. This further demonstrates that the accelerated hydrolysis phenomenon in PBT/PA6 blends results not solely from PA6 oligomer terminal amine catalysis of PBT hydrolysis, but rather from a physicochemical synergistic effect involving both “etching acceleration” and “chemical catalysis”.

## 4. Conclusions

Degradation performance assessments were conducted on industrial polymer materials exhibiting potential high-temperature hydrolysis properties, including aromatic polyesters, polyamides, and epoxy resins. This yielded a library of material platforms with controllable degradation rates across a broad range, providing data support for subsequent related work.

The degradation products of PBT and PA6 were analyzed to reveal their degradation mechanisms. Under hydrothermal conditions, the hydrolysis of the ester bond in PBT produced tetra-hydrogen furan rather 1,4-butanediol. Hydrolysis of PA6 produced caprolactam.

Inspired by the natural corrosion process of wood, PBT/PA6 blends were prepared via a simple blending process. The synergistic effect of “etching acceleration-chemical catalysis” in the blends considerably enhances the degradation rate, showing significant improvement compared to pure PBT and PA6 materials. This provides a novel direction for designing high-temperature temporary plugging agents.

## Data Availability

The original contributions presented in this study are included in the article/Supplementary Material. Further inquiries can be directed to the corresponding author.

## Supplementary Materials

The following supporting information can be downloaded at: https://www.mdpi.com/article/10.3390/polym17233090/s1. Figure S1: DSC curve of the pristine PET sample and that annealed at 180 °C for 24 h; Figure S2: Time-Weight loss plots of the pristine PET and that annealed at 180 °C for 24 h; Figure S3: DSC curves of PET samples with different heat treatment levels and hydrolysis time; Figure S4: The gas chromatogram of PBT liquid phase product and the mass spectra of PBT liquid phase products; Figure S5: The gas chromatogram of PA6 liquid phase product and the mass spectra of PA6 liquid phase products. Table S1: Melt enthalpy and crystallinity before and after hydrolysis of different PET samples; Table S2: R^2^ values of linear fits for polymers across different degradation time periods.

## References

[1] Wang J.-W., Kang Y.-Z., Zhang D.-W., Feng D.-J., Chen G., Tian L.-Y. (2021). Advances in the application of temporary plugging agents for fracturing in unconventional reservoirs. Spec. Oil Gas Reserv..

[2] Li S.-B., Wang H.-T., Ouyang S.-L. (2021). Review of research progress on chemical temporary plugging agents for oil wells. Chem. Fiber Text. Technol..

[3] Liu C., Zou H.-J., Wang Y.-G., Zhu M.-J., Su G.-S., Huang Z.-X., Yu X.-R., Yang H. (2024). Degradation behavior and mechanism of P(AM/AA/AMPS) @PLA core-shell self-degrading temporary plugging agent. J. Mol. Liq..

[4] Li J., Xiong Y., Zhang Y.-D., Lan K. (2023). A novel self-healing and degradable plugging material for high temperature gas well. J. Mol. Liq..

[5] Xu W.-X., Wang Y.-G., Zhou B.-B., Feng D.-J., Chen G., Tian L.-Y. (2022). Research and application of self-degradable water-soluble temporary plugging agents. Oilfield Chem..

[6] Pan Y., Wang J., Yang S.-C., Fu J.-W., Eteme Y.-L. (2023). Research progress of hydroxyethyl cellulose materials in oil and gas drilling and production Process. Cellulose.

[7] Qi T.-J., Han C.-Y., Luo P., Xiang L.-Y., Wang X.-B. (2013). Application of degradable fiber steering technology in steep-angle wells and horizontal wells in eastern Sichuan. Nat. Gas Ind..

[8] Zhang H.-W., Yan X.-M., Cui M.-Y., Liang C., Yan J., Sun Y.-P., Li H.-B., Zhang S.-Z. (2018). Degradable fiber and its application in horizontal well acid-fracturing. IOP Conf. Ser. Mater. Sci. Eng..

[9] Xu P., Yu J., Xie L.-Z. (2024). Synthesis and Evaluation of Plugging Gel Resistant to 140 °C for Lost Circulation Control: Effective Reduction in Leakage Rate in Drilling Process. Polymers.

[10] Zhang D.-J., Han X., Zhu J.-L., Yang S.-Y., Zhao W.-J. (2022). Preparation of (AA-AM-C_18_DMAAC-St) quaternary copolymerization temperature sensing gel microspheres by antiphase micro emulsion polymerization. Mater. Rep..

[11] Leng G.-Y., Yan W., Li F.-L., Wang B., Yuan G.-Y., Chu C.-C., Li Y.-K., Gao C.-H. (2023). Improved oil recovery in sandstone reservoirs with deep profile control technology: A comparative study between modified starch gel and polymer gel. Geoenergy Sci. Eng..

[12] Liu H.-Z., Li L.-C., Zhou M., Zheng J.-P., Wu J., Lu H.-W., Lu T., Xing L.-J., Xue H.-X. (2016). Gel performance of modified starch graft copolymer shutoff agent. Oilfield Chem..

[13] Gong W.-Z., Huang W.-A., Liu J., Zhang J.-Q., Zhang T.-Y., Jiang L., Wang Z.-B. (2021). Synthesis, characterization, and performance evaluation of starch-based degradable temporary plugging agent for environmentally friendly drilling fluid. Lithosphere.

[14] Wang R., Yan L.-S., Luo Y., Yang X.-D., Lyu B., Cheng J.-Q. (2022). High temperature plugging mechanism and experimental evaluation of temporary plugging agent. J. Petrochem. Univ..

[15] Yang S.-Q. (2021). Degradation of anhydride-cured epoxy resins and its mechanism. Contemp. Chem. Eng. Res..

[16] Wang Z.-L. (2016). Study on the Solubility, Hydrolysis, and Crystallization Behavior of Polyamide 6 Under Hydrothermal Conditions. Ph.D. Thesis.

[17] Huang M., Hu W.-Y., Song X.-Y., Liu F.-S. (2018). Ion liquid catalysis of hydrolysis reaction for waste nylon 6. Ind. Catal..

[18] Goje A.-S., Chauhan Y.-P., Mishra S. (2004). Chemical recycling, kinetics, and thermodynamics of alkaline depolymerization of waste polyesters. Polym.-Plast. Technol. Eng..

[19] Bandla S., Allahkarami M., Hanan J.-C. (2019). Thermal crystallinity and mechanical behavior of polyethylene terephthalate. Chall. Mech. Time-Depend. Mater..

[20] Luo X.-S., Huang J.-B., Zhou M., Mu X., Xu W.-W., Wu L. (2022). Theoretical study on the mechanism of hydrolysis/alcoholysis/ammonolysis of butanediol terephthalate dimer. CIESC J..

[21] Nemade A.-M., Mishra S., Zope V.-S. (2010). Chemical recycling of polyamide waste at various temperatures and pressures using high pressure autoclave technique. J. Polym. Environ..

[22] Wang Y.-J., Kong L.-F., Che H.-Y., Peng Y., Cao J.-Z. (2025). How weathering affects lignocellulose microstructure and drives brown-rot decay: Mechanistic insights. ACS Sustain. Chem. Eng..

[23] Zhang J.-Y., Zhang Y.-F. (2020). Effect of blending conditions on the structural properties of PBT/PA6 blended fibers synthetic fiber industry. New Chem. Mater..

[24] Aksit M., Groschel S., Kuhn U., Aksit A., Kreger K., Schmidt H.-W., Altstädt V. (2020). Low-density polybutylene terephthalate foams with enhanced compressive strength via a reactive-extrusion process. Polymers.

[25] Haseebuddin M.-R., Santhosh S., Shandilya A.-B. (2021). Development and characterization of PET flakes reinforced polyester resin composites. Mater. Today Proc..

