# Supplementary material for: Study on Hydrolytic Degradation of Polyester and Polyamide in Basic Solutions at High Temperatures
Source: Polymers (Basel). 2025 Nov 21;17(23):3090. doi: 10.3390/polym17233090 (PMC12693914; doi:10.3390/polym17233090)
Supplement: Supplementary file 1 [file polymers-17-03090-s001.zip › polymers-3989667-supplementary.pdf]

1. Supplementary Figures

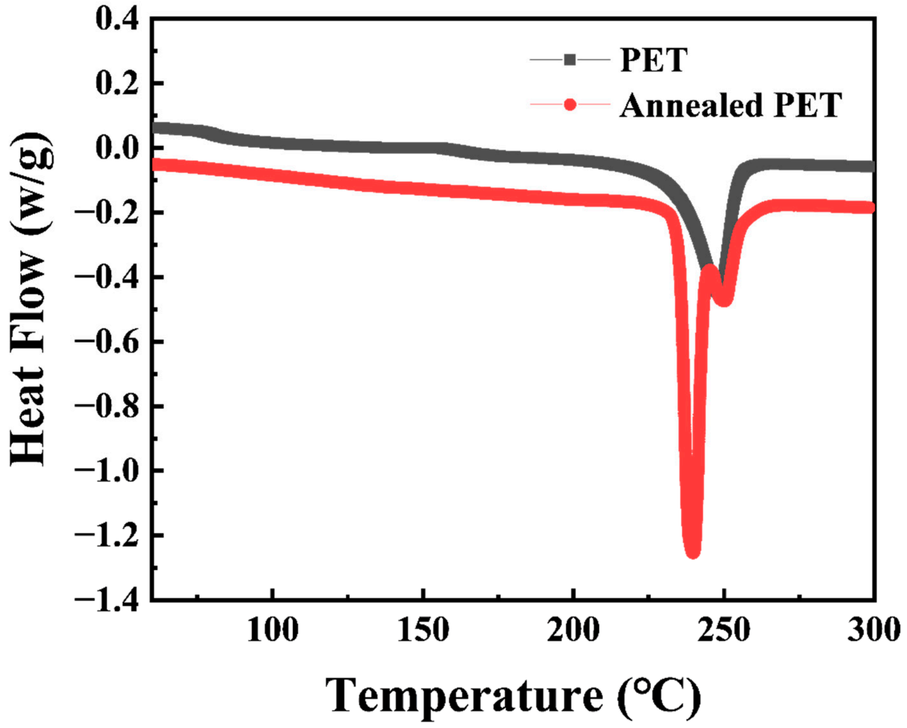

Figure S1. DSC curve of the pristine PET sample and that annealed at 180 °C for 24 h.

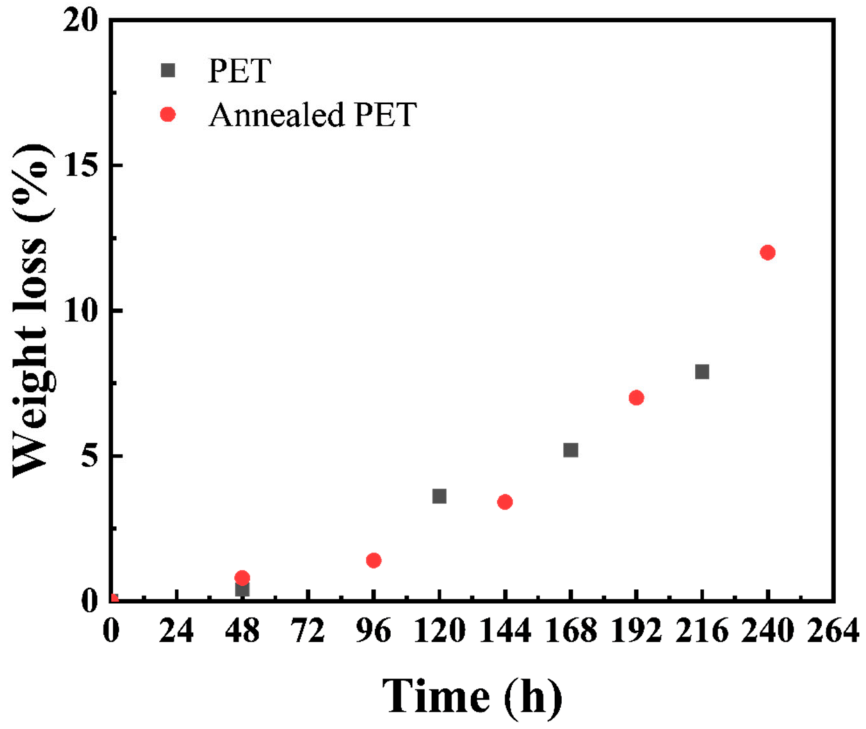

Figure S2. Time-Weight loss plots of the pristine PET and that annealed at 180 °C for 24 h.

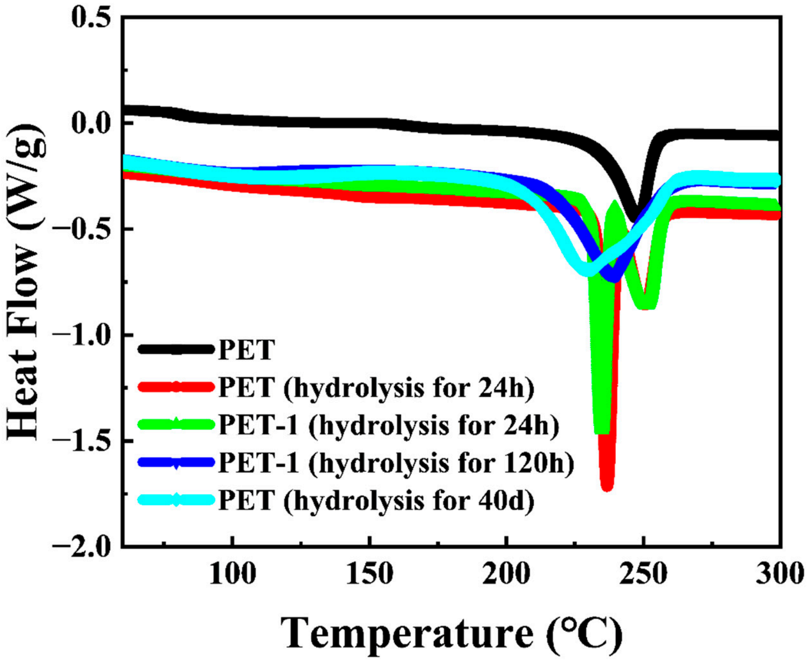

Figure S3. DSC curves of PET samples with different heat treatment levels and hydrolysis time.

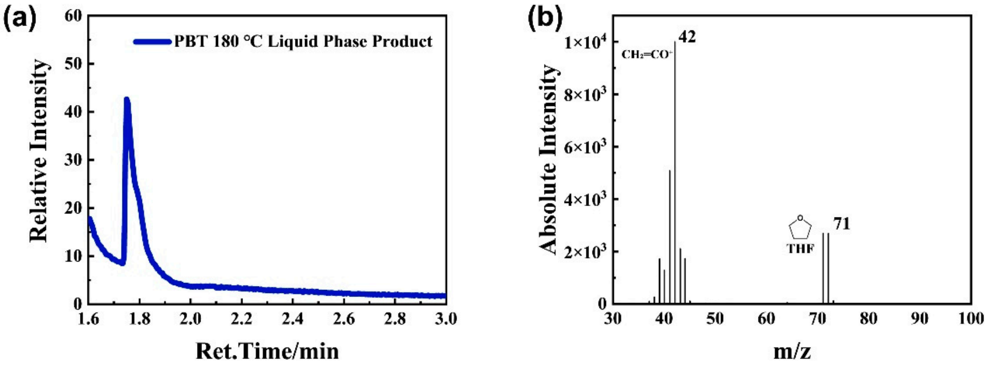

Figure S4. The gas chromatogram of PBT liquid phase product and the mass spectra of PBT liquid phase products.

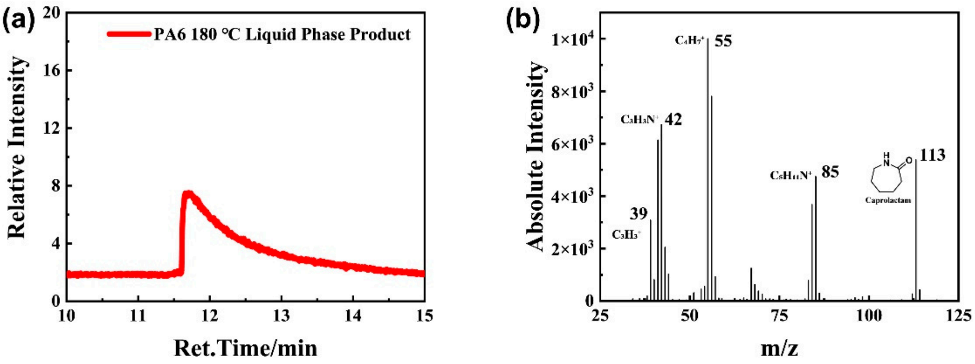

Figure S5. The gas chromatogram of PA6 liquid phase product and the mass spectra of PA6 liquid phase products.

Table S1. Melt enthalpy and crystallinity before and after hydrolysis of different PET samples.

Samples	Melt enthalpy m,J/g	Crystallinity
Non-treated PET (before hydrolysis)	36.2	26.0 %
Annealed PET	59.1	42.3 %
Non-treated PET (hydrolysis for 24 h)	64.7	46.4 %
Annealed PET (hydrolysis for 24 h)	62.2	44.6 %
Non-treated PET (hydrolysis for 120 h)	76.9	55.2 %
Annealed PET (hydrolysis for 40 d)	93.1	66.8 %

Table S2. R^2 values of linear fits for polymers across different degradation time periods.

Time \ R^2	PBT	PA6	PBT/PA6 (8/2)
0-10	0.957	0.954	0.982
11-20	0.984	0.908	0.990
21-35	0.887	0.939	0.861
